# Climatic, physical, and biogeochemical changes drive rapid oxygen loss and recovery in a marine ecosystem

**DOI:** 10.1038/s41598-019-52430-z

**Published:** 2019-11-06

**Authors:** Jesse Wilson, Gerda Ucharm, J. Michael Beman

**Affiliations:** 1 Life and Environmental Sciences, University of California Merced, Merced, CA 95343 USA; 2 Scripps Institute of Oceanography, University of California San Diego, La Jolla, CA 92037 USA; 3 Coral Reef Research Foundation, Koror, 96940 Palau

**Keywords:** Ecology, Environmental sciences, Ocean sciences

## Abstract

Dissolved oxygen (DO) concentrations shape the biogeochemistry and ecological structure of aquatic ecosystems; as a result, understanding how and why DO varies in space and time is of fundamental importance. Using high-resolution, *in situ* DO time-series collected over the course of a year in a novel marine ecosystem (Jellyfish Lake, Palau), we show that DO declined throughout the marine lake and subsequently recovered in the upper water column. These shifts were accompanied by variations in water temperature and were correlated to changes in wind, precipitation, and especially sea surface height that occurred during the 2015–2016 El Niño-Southern Oscillation event. Multiple approaches used to calculate rates of community respiration, net community production, and gross primary production from DO changes showed that DO consumption and production did not accelerate nor collapse; instead, their variance increased during lake deoxygenation and recovery, and then stabilized. Spatial and temporal variations in rates were significantly related to climatic variability and changes in DO, and causality testing indicated that these relationships were both correlative and causative. Our data indicate that climatic, physical, and biogeochemical properties and processes collectively regulated DO, producing linked feedbacks that drove DO decline and recovery.

## Introduction

Oxygen is fundamental for all organisms, yet many ecosystems experience significant spatial and temporal variations in oxygen concentrations. The factors driving oxygen variability are not well understood—variations in production via photosynthesis, respiratory consumption by a wide range of organisms, and multiple physical processes and properties (e.g., gas exchange with the atmosphere, stratification, and mixing) may be important to varying degrees. Many aquatic ecosystems are devoid of dissolved oxygen (DO) at depth due to a combination of isolation of sub-surface water from the atmosphere and community respiration (CR) of organic matter in water out of contact with the atmosphere^1,2^. Natural and anthropogenic environmental changes can also affect the biology and physics of oxygen cycling and dynamics. Warming, for instance, can lead to changes in gross primary production (GPP) and CR^3–6^, as well as changes in stratification and circulation^7,8^, that lead to oxygen loss. Determining how these and other types of change affect DO availability in aquatic ecosystems depends upon detailed understanding of interactions and feedbacks between the physical and biological processes that regulate DO^2,8^.

**Figure 1:**
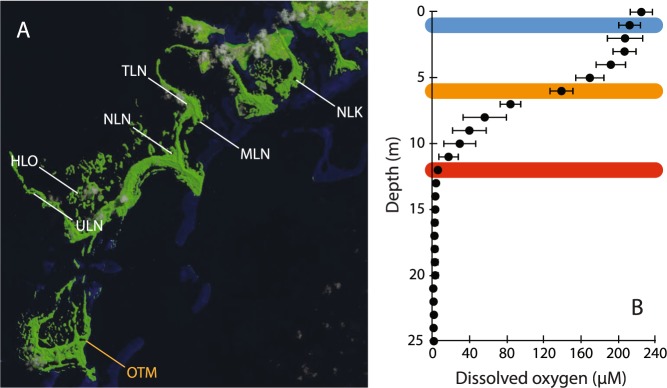
(**A**) Landsat 8 Operational Land Imager (OLI) Natural Color image of Palau collected in June 2016 and visualized with the USGS GloVis (https://glovis.usgs.gov/), with sampled marine lakes labeled. OTM is highlighted in orange at the bottom of the image. Sampled lakes had concurrent sensor- and bottle-based incubations in order to assess the best method of estimating metabolic rates, and to examine short-term variation using sensors. Sensors were deployed in OTM for more than a year during the 2015/2016 ENSO and into 2017. (**B**) Average dissolved oxygen depth profile and standard deviation for measurements made from 2013–2015 in OTM. Colored bars indicated the depths of sensor deployment for long-term measurements.

‘Marine lakes’ are bodies of seawater surrounded by land that formed as rising sea levels flooded inland basins at the end of the last glaciation^9,10^ and are ideal ecosystems in which to examine oxygen dynamics. In particular, marine lakes vary systematically in key aspects—including pronounced gradients in DO and productivity within and across lakes^11,12^—creating natural spatial and temporal gradients in physical, chemical, and biological dynamics. Dozens of marine lakes are located in the Republic of Palau, the most notable being Ongeim’l Tketau (OTM; Fig. 1A), or Jellyfish Lake, which is typically inhabited by millions of medusa. OTM is a highly-productive meromictic marine lake^12,13^, with a sharp oxycline separating oxygenated surface waters from anoxic bottom water^13^. Permanent stratification is maintained by differences in density between overlying, lower salinity surface waters that receive freshwater runoff, and water of seawater salinity below (Fig. 1B). However, OTM differs from freshwater lakes in that seawater is exchanged with the surrounding ocean and OTM is not isolated from the sea^9,13^. OTM is also known to be highly sensitive to El Niño/Southern Oscillation (ENSO) events: the strong 1997–1998 ENSO event caused a collapse of the jellyfish population (*Mastigias sp*.) in OTM, and the warming of the lake may serve as an indicator of basin-wide ENSO events^14,15^.

ENSO is a well-known periodic equatorial Pacific ocean-atmosphere climate phenomenon occurring every 2.5 to 7 years that causes changes in sea-surface temperature, sea-surface height, wind, and rainfall throughout the tropical Pacific^16,17^. While the intensity of ENSO has varied over geologic time^17–19^, it is a longstanding phenomenon^20,21^ and began activity similar to current intensity ~17,000 years ago^22^. ENSO has wide-ranging ecological effects that are still being discovered and studied^23–28^. In the Western Tropical Pacific Ocean, trade winds weaken, the western Pacific warm pool migrates eastward, and the thermocline shoals, leading to drought, higher air temperatures, lower sea surface temperatures, and lower sea surface levels^29^. Given the distinct ENSO-driven climatic changes that occur in the Western Pacific, and ecological changes in OTM observed during previous ENSO events^14,15^, we expected that climatic changes that occur during ENSO may exert strong effects on temperature, DO concentrations, CR, GPP, and/or Net Community Production (NCP, the balance between CR and GPP) in OTM. In particular, we hypothesized that changes in precipitation, winds, and sea surface height might affect the physical structure of OTM, leading to subsequent biogeochemical feedbacks that affect DO.

To provide insight into the interacting biological and physical processes that collectively affect oxygen in aquatic ecosystems, as well as their sensitivity to environmental perturbations, we examined DO concentrations in OTM using high frequency *in situ* measurements for more than one year (February 2016–March 2017)—capturing the peak of the 2015–2016 El Niño event and its decline. DO data were combined with temperature data, meteorological data, and data from shorter deployments in other lakes to quantitatively compare and contrast biological and physical controls on oxygen on multiple timescales. We observed deoxygenation of the lake and subsequent recovery, and show that this is driven by a combination of physical and biological processes.

## Results and Discussion

### Variations in oxygen and temperature

**Figure 2:**
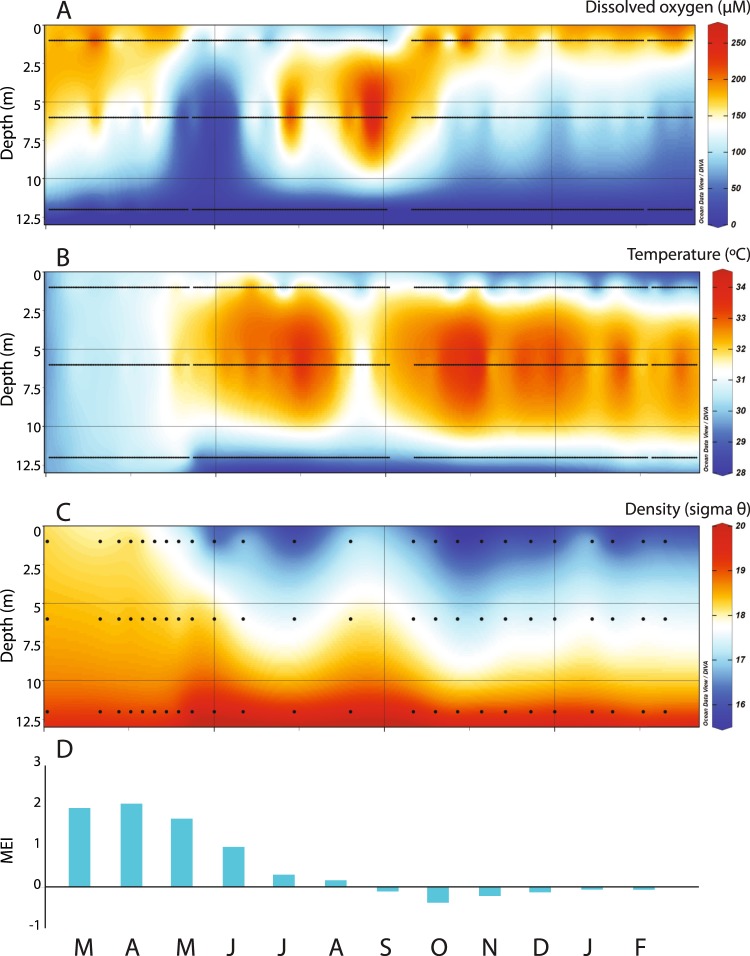
Contour plots of daily averaged (**A**) dissolved oxygen (DO) and (**B**) temperature in OTM, with dots denoting dates on which daily averages were calculated at 1, 6, and 12 m. Breaks in the time-series represent periods of time where sensors were removed from the lake for data download and battery replacement. (**C**) Density contour plot with dots denoting the dates that salinity was collected and therefore the dates that density was calculated based on salinity and temperature. (**D**) Corresponding monthly mean ENSO Index (MEI) values. Months are noted at the bottom of the figure, from March 2016 to February 2017.

Pronounced variations in DO (Fig. 2A) and temperature (Fig. 2B) occurred in OTM over the course of the time-series. DO concentrations were similar at 1 and 6 m at the start of the study, but DO gradually and then sharply declined at all three depths (1, 6, and 12 m). DO showed the greatest relative decrease at 6 m and reached an all-time low for the time-series during May 2016 (Fig. 2A). DO concentrations <40 µM—which are fatal for many metazoans^7^—were observed for nearly a month at 6 m (Fig. 2A). DO was present at 12 m at the beginning of the study but declined to undetectable levels (Fig. 2A) for nearly a year. These decreases in DO likely represent a strong perturbation to the lake ecosystem as a whole, as the oxygenated volume of the lake was substantially reduced for several months.

**Figure 3:**
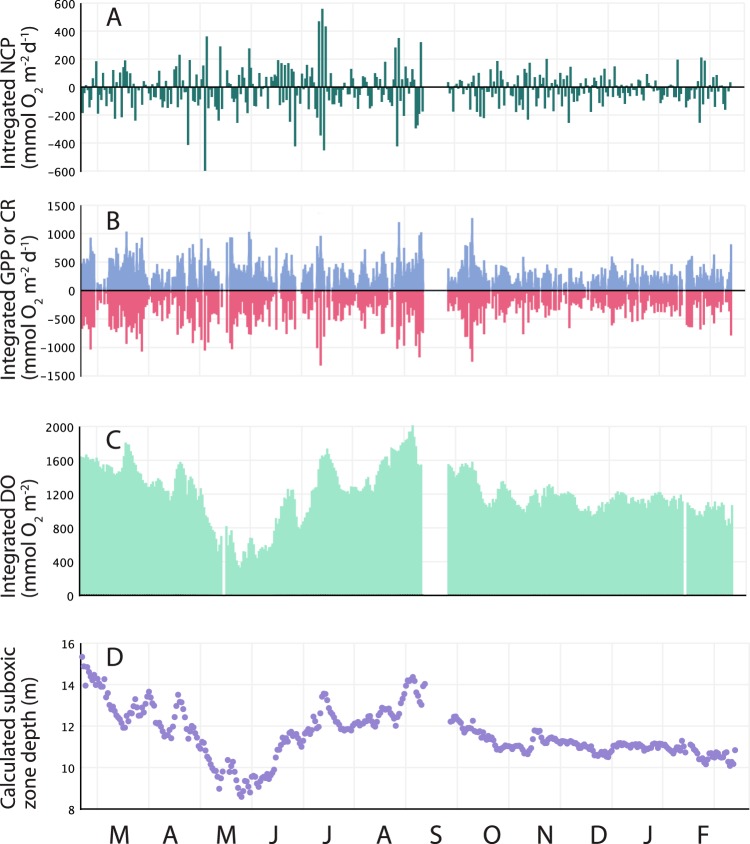
Depth-integrated (**A**) NCP and (**B**) GPP (blue; above the line) and CR (red; below the line) over time in OTM (based on measurements at 1, 6, and 12 m). (**C**) Integrated lake DO concentrations calculated via trapezoidal integration, and (**D**) suboxic zone depth calculated from DO concentrations. Months are noted at the bottom of the figure, from March 2016 to February 2017.

DO concentrations then rapidly increased at 6 m—surpassing concentrations at 1 m for two months (Fig. 2A)—but subsequently decreased to lower levels. DO concentrations at 1 m then increased in September 2016, while DO values at 12 m remained below detection until 2017. We used these data to calculate integrated DO concentrations (Fig. 3C), as well as variations in the depth of the suboxic zone (Fig. 3D; defined as the depth at which DO reaches 20 µM; ref.^30^); both calculations illustrate significant variations in DO. In particular, the suboxic zone shoaled from >14 m depth in February 2016 to <9 m in May 2016. The suboxic zone re-deepened and eventually stabilized around 11 m. While 11 m has been a common depth for the suboxic zone in OTM in recent years^11,12^, *in situ* data indicate that this is an unstable feature.

In concert with DO, lake temperatures showed substantial variation over the time-series. Most notably, all depths warmed over the same time period. At 6 m, temperatures increased to >32 °C over the initial period as the lake deoxygenated, at which point the depth of the suboxic zone correlated strongly with water temperature (P < 0.001; r^2^ = 0.87; Fig. 2B). Temperatures at 6 m continued to warm even after DO concentrations began to increase, and only began cooling in late July 2016; however, cooling was short-lived, and temperatures warmed to >32 °C by early September 2016. Temperatures at 6 m remained high throughout the remainder of the time-series and exceeded temperatures at 1 m by >2 °C (Fig. 2B).

**Figure 4:**
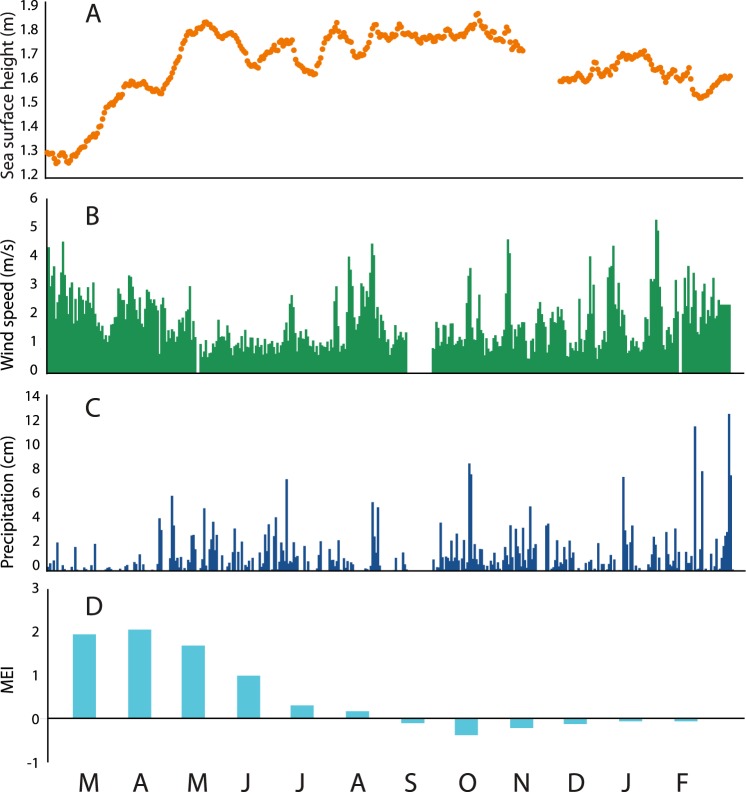
Daily time-series of (**A**) sea surface height (SSH), (**B**) wind-speed from a weather station moored in OTM, and (**C**) daily precipitation data for Koror, Palau (blue) from the National Weather Service. (**D**) Corresponding monthly mean ENSO Index (MEI) values. Months are noted at the bottom of the figure, from March 2016 to February 2017.

Temperatures at 1 m closely tracked 6 m in the beginning of the study and then diverged: temperatures remained more moderate at 1 m compared with 6 m and were punctuated by several periods of rapid cooling. Surface water temperatures in OTM are frequently lower due to the influence of freshwater input from the surrounding watershed; the evident lack of precipitation in Palau in February–April 2016 (Fig. 4C) is consistent with patterns observed across the Western Pacific during El Niño, and likely produced parallel temperature trends at 1 and 6 m due to reduced freshwater input during the beginning of the time-series. This observation is reinforced by direct, but infrequent, salinity measurements (Fig. S1) that allowed us to calculate water density (Fig. 2C). These data showed higher salinities and densities at 1 and 6 m during the early part of the time-series (Fig. 2C). During this time (February–March 2016) stratification was reduced, the suboxic zone deepened, and rainfall was at its lowest (Fig. 4C). 1 and 6 m temperature (Fig. 2B) and density (Fig. 2C) trends then diverged as precipitation increased, with cooling episodes at 1 m corresponding with higher wind-speeds and precipitation associated with storms (Fig. 4B,C; in July, August, and October 2016, and in January 2017, for example). At 12 m, temperatures increased then rapidly cooled, and then displayed a steady increase through time (Fig. 2B).

MEI—a bimonthly value that estimates ENSO from the first principal component of six different ocean-atmosphere measurements—ranged from >2.0 at the start of the study in February 2016—indicating a strong El Niño event—to <0 by September 2016—indicating the termination of El Niño (Figs 2D, 4D). Given the strength of the 2015–2016 ENSO event, and the documented ecological effects of ENSO in OTM^14^, we examined regional climate changes associated with ENSO that might affect DO and temperature in OTM, and that are captured by daily data: air temperature, wind-speed, precipitation, and sea surface height (SSH) may all affect the physical properties of the lake. In general, air temperature, wind-speed, and precipitation significantly (P < 0.001–0.03) but weakly (r^2^ = 0.008–0.19) correlated with lake DO and water temperature at various depths—with wind-speed acting as the strongest environmental control on temperature at 1 m (r^2^ = 0.19) and DO at 12 m (r^2^ = 0.12). Precipitation was lower at the beginning of the study, which affected water column salinity (and density)—such that salinity and density (only analyzed on dates salinity was collected) closely followed MEI at 1 m (P < 0.01, r^2^ = 0.816–0.777). SSH was lowest at the beginning of the study and increased as the suboxic zone shoaled and integrated DO declined (P < 0.001, r^2^ = 0.790–0.768; Fig. 4A), correlating strongly with daily DO at 1 and 12 m (P < 0.001, r^2^ = 0.226–0.589).

Observed changes in DO were therefore significantly related to several environmental changes. When SSH and precipitation (and thus freshwater input) were low at the beginning of the study, density stratification occurred at greater depths. However, stratification increased along with SSH, rainfall, and surface heating, causing the suboxic zone to shoal. The increase in SSH, in particular, likely increased the volume of seawater in the lake. However, these changes did not explain all of the variation in integrated DO and suboxic zone depth, especially as the lake re-oxygenated. As a result, we examined whether the biological processes that produce and consume oxygen (GPP and CR) also affected DO in Jellyfish Lake, and whether these processes were related to environmental changes. We also assessed how ΔDO—the change in DO between sequential days—varied with metabolic rates and environmental changes—in order to determine the degree to which air-sea flux, variations in SSH, and biological activity affected water column oxygen content.

### Biogeochemical rate variations

Given the distinct patterns in DO time-series that were observed at each depth, we analyzed both integrated and depth-specific rates of CR, NCP, and GPP calculated via several approaches on a daily timescale. Integrated rates (Fig. 3A,B) showed that NCP was not consistently autotrophic or heterotrophic (Fig. 3A); instead, NCP displayed wide variation as the lake deoxygenated and re-oxygenated in mid-2016, and was generally less variable later in the time-series as stratification stabilized. Integrated CR was also variable but generally elevated as the lake deoxygenated and re-oxygenated (Fig. 3B). The combination of high CR values with variable NCP led to the highest rates of integrated GPP (Fig. 3B). CR was significantly correlated with GPP (r^2^ = 0.783) with a slope of 0.950 and intercept of 35.2, indicating net autotrophy. This is similar to work by Staehr and Sand-Jensen^31^, who found that CR increased with GPP in a seasonal freshwater lake.

We also examined relationships between integrated rates and environmental data, and found that CR and GPP were weakly, significantly, and similarly correlated with integrated DO (Fig. 3B,C), water temperature, and precipitation (Table 1). In contrast, integrated NCP was weakly correlated with air temperature and precipitation. A notable feature of these data are short periods where integrated DO increased sharply (Fig. 3C), and the suboxic zone deepened (Fig. 3D); because rates are calculated from variations in DO, these are also periods of high calculated NCP and GPP (see below). Both rates also correlated with changes in integrated DO (ΔDO), with the strongest correspondence between integrated NCP and ΔDO (r = 0.505; Table 1). This is indicative of a positive relationship between metabolic balance and changes in water column oxygenation.

**Table 1:** r-values for significant relationships (P < 0.05) between CR, GPP, NCP, and environmental variables.

Rate	DO	Water Temperature	Air temperature	Precipitation	Wind-speed	Sea Surface Height	ΔDO
**Integrated**							
CR	0.198	−0.185		−0.156			
GPP	0.212	−0.181	0.159	−0.239			0.289
NCP			0.184	−0.114			0.505
**1** **m**							
CR		0.214	0.103		−0.244		
GPP	0.184	0.213			−0.302	0.107	0.306
NCP	0.179						0.389
**6** **m**							
CR	0.174	−0.274		−0.122			
GPP	0.359	−0.258	0.141	−0.209			0.329
NCP	0.170						0.335

Environmental variables for the depth at which a rate took place were used. When a depth integrated rate was assessed we utilized the depth integrated environmental variables (using trapezoidal integration).

Integrated rates incorporate changes in DO, as well as calculated fluxes of oxygen into or out of the lake (depending on oxygen saturation; see Methods). We found that DO was consistently undersaturated at 1 m throughout the study (average %DO = 84.8%), such that oxygen was nearly always fluxing into the lake (Fig. S2A). However, the contribution of air-water oxygen flux to overall rates was minor, with a mean value of 2.14% and a median value of 0.28% (Fig. S2B). Although this flux is calculated from windspeed relationships, integrated rates were not significantly related to wind-speed (Table 1). At the whole lake level, then, changes in integrated DO are mostly strongly related to biological activity rather than air-water fluxes of oxygen.

In order to make depth-specific comparisons between rates, environment variations, and ΔDO, we first determined the best approach for calculating rates at each depth. This was achieved by comparing *in situ* sensor-based rate calculations with bottle incubations in both meromictic and holomictic lakes (unstratified lakes with lower nutrient concentrations throughout). We found that the slope approach^32^ for calculating CR and NCP from the oxygen sensor data at each depth was the most similar to *in situ* bottle incubations (r^2^ = 0.499 and P = 0.024 for NCP; r^2^ = 0.311 and P = 0.09 for CR [n = 10]; Fig. S3). Notably, rate magnitudes were similar between sensors and bottles (Fig. S3) and consistent with earlier work showing high rates of GPP^13,12^ and CR^12^. Over multi-day sensor deployments in each lake, surface NCP values were frequently autotrophic (NCP >0) while deeper depths in holomictic lakes were often heterotrophic (NCP <0), reflecting a decline in GPP with depth. However, the meromictic lakes (OTM, NLK, and TLN) all typically displayed subsurface maxima in both CR and GPP located near the suboxic zone in each lake. Because rates of both processes were often high, their balance often shifted from day to day, leading to variable NCP values. These data are suggestive of dynamic oxygen cycling in the meromictic lakes, including OTM.

**Figure 5:**
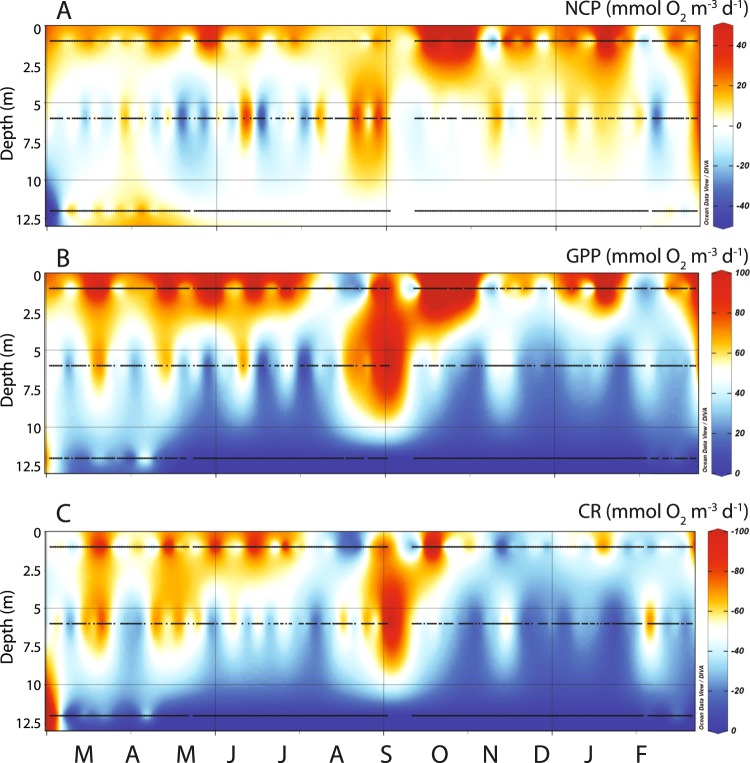
Contour plots of daily (**A**) NCP, (**B**) GPP, and (**C**) CR, with dots denoting dates on which rates were calculated at 1, 6, and 12 m. Values were calculated using the Slope Approach (see Methods) and are plotted on a common vertical axis to illustrate differences. Months are noted at the bottom of the figure, from March 2016 to February 2017.

We applied the slope approach to our long-term oxygen data to calculate CR, NCP, and GPP at each depth (Figs 5, S4). However, due to the consistent lack of DO at 12 m, metabolic rates were obviously only greater than zero prior to the loss of DO and at the end of the time-series; as a result, we did not analyze rates at 12 m in comparison with environmental data. (Organic material may also be respired anaerobically at 12 m, and anoxygenic photosynthesis may contribute to overall production in OTM, but our focus was on CR, NCP, and GPP as modulators of DO that can be measured *in situ* at high resolution). At 1 m, NCP values were mostly positive throughout the time-series—making this depth a net source of DO—and reached higher values during the last half of the time-series (Figs 5A, S4A,B and S5A,B). NCP values at 6 m were more commonly negative in the early part of the time series, leading to this layer being a net sink for DO at certain times (Figs 5A, S4C,D and S5C,D). NCP at 6 m then increased in magnitude and oscillated as the lake re-oxygenated in June of 2016, and then shifted to being roughly in balance during the last half of the time-series. Variance in time-series signals may be indicative of phase shifts as a system ‘flickers’ between states^33,34^, and increased variance has been identified in freshwater lakes undergoing ecological transitions^35^. CR, NCP, and GPP all displayed high variance prior to lake deoxygenation and recovery (Figs 5, S4 and S5). At 6 m, the highest variance in CR, NCP, and GPP preceded the drop in DO while variance at 1 m, while also high during this time, was greatest as the lake recovered (Fig. S4).

NCP reflects the balance between GPP and CR, and changes in NCP at 1 m were driven both by higher GPP and lower CR values later in the time-series (Figs 5, S4A,B and S5A,B). However, CR is often tightly coupled to GPP in marine systems^6,36^, and our work reinforces this concept, as overall CR positively correlated with GPP at both 1 m (P < 0.001, r^2^ = 0.483) and 6 m (P < 0.001, r^2^ = 0.260). At 1 m, both CR and GPP were positively correlated with water temperature, which has been observed in both the ocean^3,6^ and estuaries^37^. CR and GPP at 1 m were also negatively correlated with wind-speed, while GPP was positively correlated with SSH, and GPP and NCP were correlated with DO. At 6 m, CR, NCP, and GPP were more variable during the initial deoxygenation, and rates decreased as the 2016 El Niño concluded in the summer (Figs 5, S4C,D and S5C,D). All were positively correlated with DO, while CR and GPP also negatively correlated with water temperature and precipitation (Table 1). Similar to depth-integrated GPP and NCP, depth-specific GPP and NCP were correlated with ΔDO (Table 1). Patterns at individual depths are therefore consistent with those at the whole-lake level, and show generally weak relationships between biogeochemical rates and the physical variables (temperature, precipitation, wind, SSH), whereas rates were consistently correlated with DO and changes in DO (ΔDO).

### Statistical tests for causality and multivariate analysis

The data and univariate analyses above indicate that changes in lake DO concentrations do not have a single clear physical or biological driver; instead, we hypothesized that a series of linked feedbacks lead to deoxygenation and recovery in OTM. We used two main approaches to identify these potential feedbacks, including (a) tests of causality and (b) a machine learning approach that incorporates multiple types of data into a multivariate framework.

To determine the directionality of the relationships identified above, we used Granger Causality Tests with a lag of one day to assess whether certain variables forecasted changes in other variables—with a particular focus on whether the biogeochemical rates forecasted changes in DO. Despite relatively weak regression statistics, wind was the main physical factor that played a role in forecasting changes in other physical, chemical, and biological variables. At 1 m, wind forecasted changes in GPP (P = 0.014) and temperature (P < 0.001); at 6 m, wind forecasted changes in DO (P = 0.003) and temperature (P < 0.001); and wind also forecasted changes in suboxic zone depth (P = 0.001). The effect of wind on stratification and water temperature is well documented, with higher wind-speeds potentially causing stratified boundaries to tilt and mix—especially when this boundary is near the surface—along with evaporative cooling and/or changes in temperature due to lake mixing^38,39^. In a large-scale lake experiment^40^, high rates of GPP were followed by high rates of CR during mixing events, leading to increased net heterotrophy following stratification. In OTM, when stratification weakened due to changes in wind, temperature, and, in our case, an increased supply of ocean water through the karst limestone rock (indicated by higher SSH), photosynthetic organisms evidently capitalized on enhanced nutrient availability—thus setting in motion an upper water column carbon/oxygen cycle. CR appears to have responded quickly and efficiently to GPP as surface DO was undersaturated. Comparison between the density contour plot and GPP plots confirm this: when stratification began to weaken in August 2016, GPP was elevated at 6 m and subsequently increased at 1 m, while NCP displayed high variability as CR responded (Figs 2C, 5).

Granger Causality Tests with a lag of one day also showed that integrated NCP caused changes integrated DO (P = 0.045), that CR caused changes in DO at 1 m (P = 0.018) and 6 m (P = 0.0042), and that CR at 6 m forecasted changes in the depth of the suboxic zone (P = 0.014). CR also forecasted changes in ΔDO at all three depths while the reverse was not true (P < 0.001–0.05). These results suggest that microbial and macrobial respiration were contributing to a drawdown in DO. Changes in DO also forecasted changes in GPP at both 6 m (P = 0.003) and 12 m (P < 0.001). The direction of this relationship is surprising, as we might expect GPP to affect DO, rather than the other way around. However, this likely reflects the presence of nutrients found in low DO water: ammonium and phosphate increase in the suboxic zone, and GPP was observed to increase with ammonium additions in OTM^12^. This idea is further supported by the fact that the depth of the suboxic zone changed before GPP changed at 6 m (P = 0.0048). As the lake deoxygenated and the suboxic zone shoaled, the signal of low DO is also likely associated with increased nutrient availability, leading to the observed effect on GPP.

Causality test results are further consistent with using boosted regression trees (BRT) to model the oxygen status of the lake—including both depth-integrated DO, suboxic zone depth, and depth-integrated changes in DO. We found that BRT captured 93.4% of the variation in integrated DO, 94.2% of the variation in suboxic zone depth, and 80% of the variation in integrated ΔDO. For integrated DO, the most informative variable was monthly MEI (34.4%), followed by SSH (23.7%), water temperature (19.4%), and then integrated GPP (7.41%); suboxic zone depth was informed by MEI (36.0%), water temperature (24.8%) and SSH (20.7%), while all other variables contributed <5%. ΔDO was best explained by a combination of NCP (37.6%), GPP (12.6%), precipitation (11.8%), wind-speed (9.09%), water temperature (7.6%), and SSH (7.3%). These results once again establish the connection between physical changes in the lake (e.g., in SSH and temperature), as well as metabolic rates, and DO/ΔDO. Indeed, Bush *et al*.^41^ found that changes in microbial activity are involved in shifts between oxic/anoxic states in a seasonally stratified lake: both models and empirical data indicated that the system abruptly shifted from oxic to anoxic (with changes in the microbial community) upon relatively small changes in water column DO (and stratification), with reoxygenation taking a much longer time to occur. This work demonstrated mathematically how small physical changes can create a tipping point around which ecological functioning generates a feedback loop—similar to what occurred in OTM, with the shoaling in suboxic zone depth followed by the gradual re-deepening.

### Biogeochemical and physical dynamics of oxygen during a phase shift

Our data and analyses provide insight into the physical and biological processes and feedbacks that regulate DO in aquatic ecosystems. Over the course of a year, OTM experienced rapid and intense deoxygenation, with oxygenated water confined to just a few meters depth in mid-2016. However, DO concentrations rapidly recovered from their decline near the surface—and did so despite persistently warm temperatures and oscillating density stratification that did not revert to normal conditions for several months following re-oxygenation—while DO remained below detection at 12 m for an extended period of time.

Results from regression, causality tests, and BRT indicate that the physical limnology of the lake was most important in governing DO at each depth (and thus suboxic zone depth), particularly during the peak of the 2015–2016 ENSO event. Decreased winds, reduced rainfall and freshwater input, and increased SSH (Fig. 4) led to lake deoxygenation (Figs 2A, 3C). Increased heating during this drier period evidently increased lake temperature and thermal stratification in the water column (Fig. 2B), while manual measurements show that surface salinity increased, leading to more similar values throughout the water column (Fig. S1). These coincident changes point to shoaling of the suboxic zone and deoxygenation in response to climactic changes.

However, variations in lake DO are less clear-cut after this time period, as integrated DO increased and the suboxic zone re-deepened while mixed layer temperatures remained warm. Many studies emphasize the importance of the physical environment in determining DO concentrations^7,8^ but if density and physical mixing (wind-speed) were the only factors that affected DO concentration, then the re-oxygenation would not have occurred as the temperature (and density) remained above normal following the peak of the 2016 ENSO event. Biological oxygen cycling may have provided several feedbacks to the initial deoxygenation event: based on correlations and causality tests, increased wind caused changes in DO (and nutrient) concentrations in deeper portions of the mixed layer, which stimulated GPP. Results from BRT are consistent with this, and suggest three phases to DO cycling in OTM: (a) a deoxygenation phase driven by lake warming linked to regional climatic changes; (b) a reoxygenation phase driven at least in part by pulses of increased primary production; and (c) a more stable DO phase. Our comparisons with direct bottle-based rate measurements (Fig. S3), and the lack of correspondence between DO and physical variables (e.g., temperature) during the reoxygenation phase, suggest that pulses of increased primary production played a partial role in lake reoxygenation. Deoxygenation is nonlinear^8^, and our results highlight oxygen production and consumption feedback mechanisms that may be present and particularly relevant in other low-oxygen aquatic ecosystems.

## Methods

### Oxygen and temperature data

MiniDOT optical dissolved oxygen and temperature sensors (Precision Measurement Engineering Inc., Vista, CA, USA) were used to measure DO and temperature at 1, 6, and 12 m in OTM at 10-minute intervals from February 18, 2016 through March 6, 2017. DO concentrations were corrected for variations in salinity based on salinity profiles that were collected throughout the year (see below), with the most recent past average salinity value for a depth used to correct DO concentrations. Dataloggers were removed on May 12, 2016, September 7, 2016, and February 6, 2017 (to collect data and have the batteries replaced) and were returned to the lake on May 13, 2016, September 20, 2016, and February 7, 2017. At those times, there was no evidence of biofouling on the sensors (copper mesh was attached to the sensors to prevent biofouling), and the data showed no drift when comparing pre-removal vs. redeployment (Fig. 2A,B). Sensor DO values also agreed closely with DO profiles taken in the lake.

### Calculations of CR, NCP, and GPP

CR, NCP, and GPP were each calculated over the time period of deployment for (a) the integrated upper 12 m and (b) for each depth. We calculated both integrated rates and rates at each depth using the approach of Needoba *et al*.^42^, a method that has been adapted for *in situ* sensors and applied in nearshore ecosystems and estuaries. Briefly, biological change in oxygen (BDO) was calculated between each logged data point (10-minute time intervals) and then summed over (a) dark hours (from 19:00 to 5:00; and then converted to a per day rate) for CR and (b) 24 hours (from 5:00 to 5:00 the following day) for NCP. GPP was calculated by adding the absolute value of CR to NCP. No GPP was calculated when CR was positive (i.e., when DO did not decrease over night). For BDO in the integrated upper 12 m, oxygen flux into and out of the water was calculated for each 10-minute change using the methods presented in Needoba *et al*.^42^, in which Schmidt numbers (with oxygen Schmidt numbers being calculated via temperature and salinity and the Schmidt number for modeled CO_2_ being 660 in saltwater)(Eqs 1, 2) are used to convert a modeled transfer coefficient for CO_2_ (k_CO2_), based on wind-speed at 10 m (U_10_)(Eq. 3), to a diffusion coefficient for oxygen (Eq. 4). The transfer coefficient for oxygen (k_O2_) is then multiplied by the difference of DO and DO at saturation to obtain the oxygen flux (Eq. 5). DO at saturation was calculated by the MiniDOT datalogger software, which uses elevation above sea level, temperature, and salinity. The conversion of Schmidt numbers to gas transfer coefficients was done based on the experiments by Jahne *et al*.^43^ in which n = −2/3 if wind-speed at 10 m is less than 2 m/s or −1/2 if wind-speed at 10 m is greater than or equal to 2 m/s (Eq. 4).

1 $$S{c}_{0}=1800.6-120.10T+3.7818{T}^{2}-0.047608{T}^{3}$$

2 $$S{c}_{O2}=S{c}_{0}(1+3.14\times {10}^{-3}S)$$

3 $${k}_{CO2}=0.31\times {({u}_{10})}^{2}$$

4 $$\frac{{k}_{O2}}{{k}_{CO2}}={(\frac{S{c}_{O2}}{660})}^{n}$$

5 $${F}_{O2}=-\,{k}_{O2}(DO-D{O}_{sat})$$

Depth-specific CR, NCP, and GPP were also calculated two other ways: (a) by using a slope approach^32^ and (b) by using spectral analysis to remove the tidal cycle before applying the slope approach. For the slope approach, the slope of DO concentrations versus time during dark hours (19:00 to 5:00), was used to calculate daily CR for each depth. Daily NCP for each depth was calculated from the slope of DO concentrations versus time over a full 24-hour cycle. Rather than include portions of one night, followed by daytime hours, and then portions of another night, the preceding day was paired with the following night—effectively reproducing the 12 hour light/dark cycle in the tropics. Daily GPP was calculated by adding the absolute value of daily CR to daily NCP. For spectral analysis, the “Rssa” package in R was used; variation in the 10-minute DO concentrations was deconstructed and then reconstructed using only the daily cycle (without the tidal flux or small-scale variations) for each depth. Following this, the slope approach was used as described above. Spectral analysis proved extremely useful at 12 m from May 14, 2016 to September 6, 2016 when there was an enhanced tidal cycle that made the regular slope approach infeasible. The outliers to the multi-lake analysis (Fig. S3) represented holomictic lakes with enhanced tidal cycles, reinforcing the use of spectral analysis to remove the tidal signal when an enhanced tidal cycle was observed at 12 m in OTM. During the sensor deployment period from September 21, 2016 to February 5, 2017, a consistent lack of DO at 12 m rendered both spectral analysis and the slope approach unusable.

### Comparison with bottle-based measurements

We tested the reliability of calculating CR and NCP via *in situ* sensors through multiple concurrent deployments of *in situ* bottle experiments in different marine lakes^12^. These comparisons included both meromictic (stratified) lakes: Ngermeuangel Lake (abbreviated NLK), Ongeim’l Tketau (OTM aka Jellyfish Lake), and T Lake (TLN); and holomictic (mixed) lakes: Heliofungia Lake (HLO), Mekeald Lake (MLN), Ngeruktabel Lake (NLN), and Uet era Ngchas (ULN). Triplicate clear 300 mL Wheaton BOD bottles were used to measure NCP, while triplicate black Plastisol-coated 300 mL Wheaton BOD bottles were used to measure CR^12^. Bottles were filled to at least two times overflowing via slow laminar flow in order to exclude bubbles and incubated at collection depth in each lake on a floating array. This allowed us to assess which method of estimating a depth specific CR, NCP, and GPP was most accurate on a daily scale (BDO, Slope Approach, or Spectral Analysis with the Slope Approach).

### Environmental data

To assess potential variations in the depth of the suboxic zone in OTM, we used DO measured by the three sensors in the lake to calculate an expected suboxic zone depth. Linear regression splines were fit to the average DO values measured at each depth, and we solved for the depth at which the oxygen concentration reached 20 µM—a value frequently used to define low-oxygen marine ecosystems^30^. This approach overestimated the depth of the suboxic zone for two periods of time in July and August-September, as the estimated suboxic depth exceeded 12 m, but no DO was present at 12 m. At these times, DO concentrations at 6 m exceeded those at 1 m, which likely led to the overestimation. However, this is just one of several approaches we used to examine DO variations in OTM over time. In addition to depth-specific DO (and temperature), we calculated ΔDO (changes in DO between days), integrated DO concentrations, integrated ΔDO, and integrated temperature via trapezoidal integration across the different depth layers in the lake.

Salinity profiles were collected using a Hydrolab DS5 water quality sonde (OTT Hydromet, Sheffield, UK) every two weeks to one month at two different sites in OTM and then averaged to obtain one salinity measure for each depth of interest. *In situ* density was calculated on dates that salinity was collected based on temperature and salinity^44^. Wind-speed and direction were collected using a 05106 Wind Monitor (R.M. Young Company, Traverse City, MI, USA), mounted 3 m above OTM lake surface every 15 minutes throughout the study period and matched to DO timepoints. Winds were predominantly Easterly and were binned into 8 categories in order to test for significance with ΔDO and metabolic rates; but only GPP at 1 m weakly correlated with wind direction (P = 0.002; r^2^ = 0.055). Wind-speed was converted to 10 m above lake surface using the equation provided by Donelan^45^ in order to estimate the flux of oxygen into and out of the water column. When daily values of CR, NCP, and GPP were utilized for analysis, DO, temperature, wind-speed, and wind direction were averaged for each 24-hour period (from 5:00 to 5:00 the following day) to relate them statistically. Similarly, data were averaged on an hourly timescale when compared with hourly measurements (data not shown). Daily mean air temperature and daily precipitation were collected via the Koror Weather Service (Station: GHCND:PSW00040309; 7.33333°N, 134.48333°E) and downloaded from the National Oceanic and Atmospheric Administration National Centers for Environmental Information (https://www.ncdc.noaa.gov/cdo-web/datasets/GHCND/stations/GHCND:PSW00040309/detail; Order ID: 908369) on March 26, 2017. Sea surface height (SSH) was downloaded from the University of Hawaii Sea Level Center (http://uhslc.soest.hawaii.edu/data/) on July 11, 2019^46^. Finally, MEI—a bimonthly value that estimates ENSO based on six different ocean-atmosphere measurements—was obtained from the NOAA website (https://www.esrl.noaa.gov/psd/enso/mei/table.html) on April 8, 2017. Strongly positive MEI values are indicative of El Niño events while strongly negative values reflect La Niña conditions.

### Statistical analyses

Statistical analyses were conducted in R Studio (version 1.0.136). Linear regression was used for initial tests of significant relationships between different variables. All regression models satisfied generalized linear model assumptions with the exception of those involving wind-speed, which exhibited slight inhomogeneity of variance that transformations did not correct. Unless stated otherwise, we used the na.spline command in the “zoo” package when no data existed for salinity when relating variables to one another. For causative testing we used the Granger Causality test (in the “lmtest” package) to assess if certain variables predicted others on daily timescales. Variance and auto-correlation (data not shown) of daily time-series were examined using the qda-ews command in the “earlywarnings” R package with a rolling window size of 15%.

Boosted regression trees (BRT; aka gradient boosting machines) were used to analyze multivariate relationships between integrated DO and suboxic zone depth, related to environmental data (monthly MEI, daily meteorological data, and daily lake temperature values) and biogeochemical rate data (daily CR, GPP, and NCP). BRT is a machine learning approach that relies on regression trees to model a response variable based on multiple predictors, but it does so repeatedly and stochastically to build an ensemble of successive trees. BRT is useful in a case such as this, where multiple variables are likely related in a varying hierarchy over the times-series. We used the “gbm” command in the gbm R package with a bag fraction of 0.7 (which excludes 30% of the data from each tree-building step, thereby introducing stochasticity into the analysis). We varied the number of trees (100–363), shrinkage rate (0.01–0.1), and variable interaction depth^2–5^, but found only minor differences in results; we report results using 363 trees, a shrinkage rate of 0.05, and a variable interaction depth of 3. Sensitivity analyses were conducted by systematically varying the range of a variable of interest (e.g., temperature, GPP) while other variable values were fixed to their mean values over the time-series.

## Supplementary information

Supplementary Figures
