# Supplementary material for: Climatic, physical, and biogeochemical changes drive rapid oxygen loss and recovery in a marine ecosystem
Source: Sci Rep. 2019 Nov 6;9:16114. doi: 10.1038/s41598-019-52430-z (PMC6834605; doi:10.1038/s41598-019-52430-z)
Supplement: Supplementary file 1 — Supplementary Figures [file 41598_2019_52430_MOESM1_ESM.pdf]

# Climatic, physical, and biogeochemical changes drive rapid oxygen loss and recovery in a marine ecosystem

Jesse Wilson[1,2], Gerda Ucharm[3], and J. Michael Beman[1]

[1]Life and Environmental Sciences, University of California Merced, Merced, CA, 95343, USA

[2]Scripps Institute of Oceanography, University of California San Diego, La Jolla, CA, 92037, USA

[3]Coral Reef Research Foundation, Koror, Palau, 96940

Corresponding author: Jesse Wilson, Scripps Institute of Oceanography, University of California San Diego, La Jolla, CA, 92037, USA

E-mail: jessewilson13@gmail.com

Phone: +1.805.403.6464

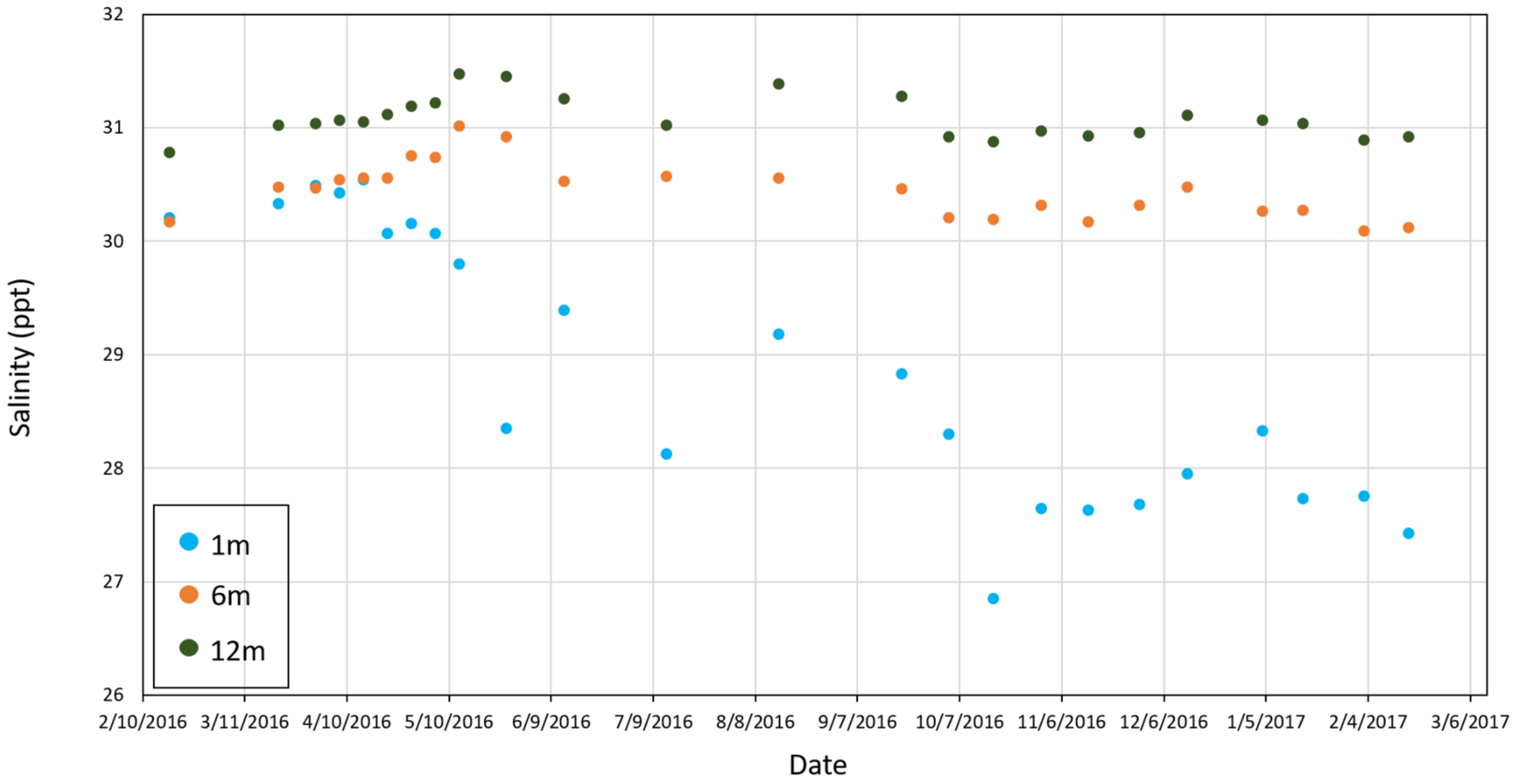


**Figure S1.** Salinity profiles were collected at 1m, 6m, and 12m in 2016 on February 18, March 21, April 1, April 8, April 15, April 22, April 29, May 6, May 13, May 27, June 13, July 13, August 15, September 20, October 4, October 17, October 31, November 14, November 29, December 13, and in 2017 on January 4, January 16, February 3, February 16. Two different sites (an Eastern site and a Western site) in the lake were measured and the value reported is the average of those values.

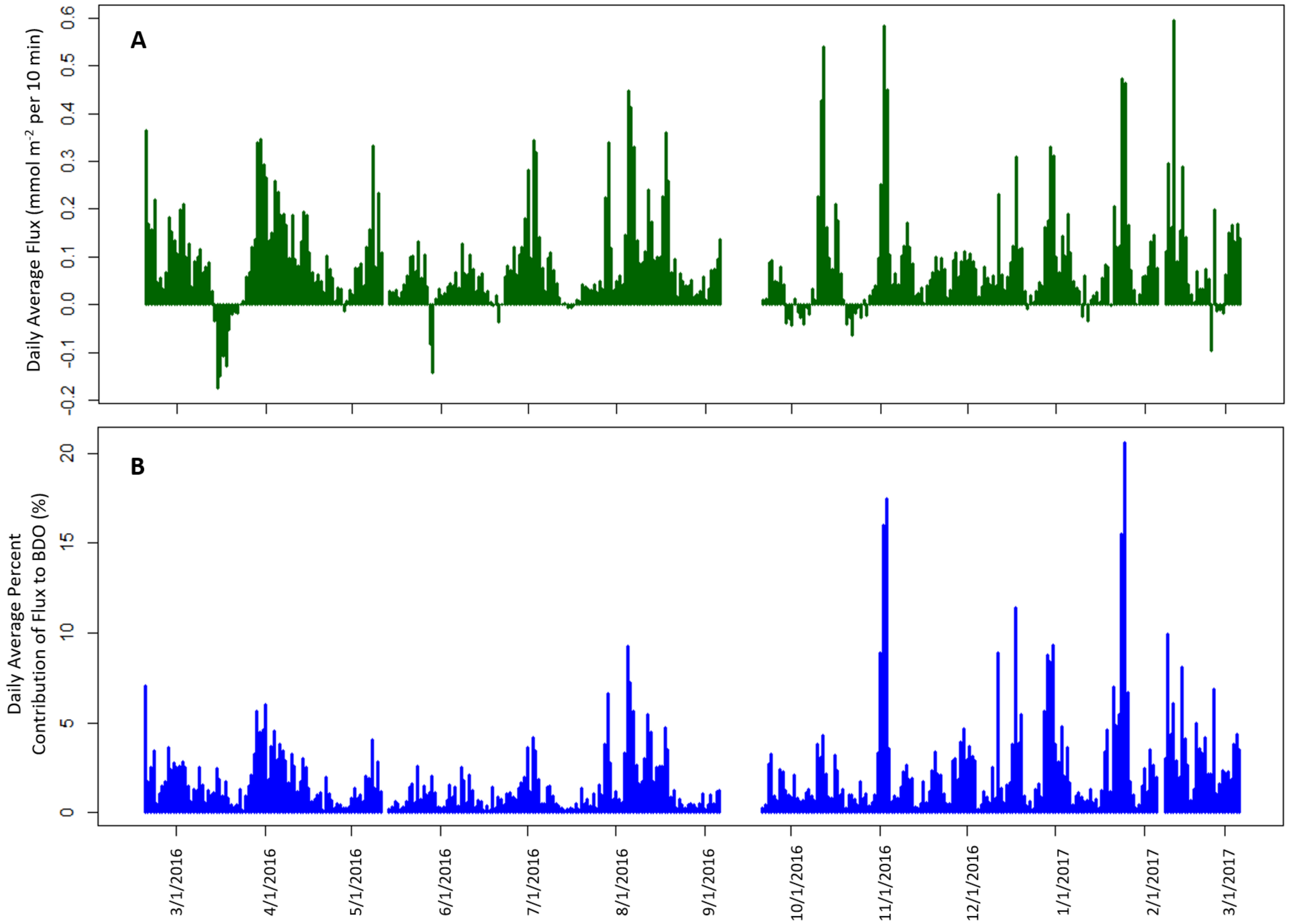


**Figure S2.** Daily averages of (A) 10-minute air-water oxygen flux that went into calculating 10-minute BDO rates (that were then summed for a daily value) and (B) percent contribution of 10-minute air-water oxygen flux to 10-minute BDO rates.

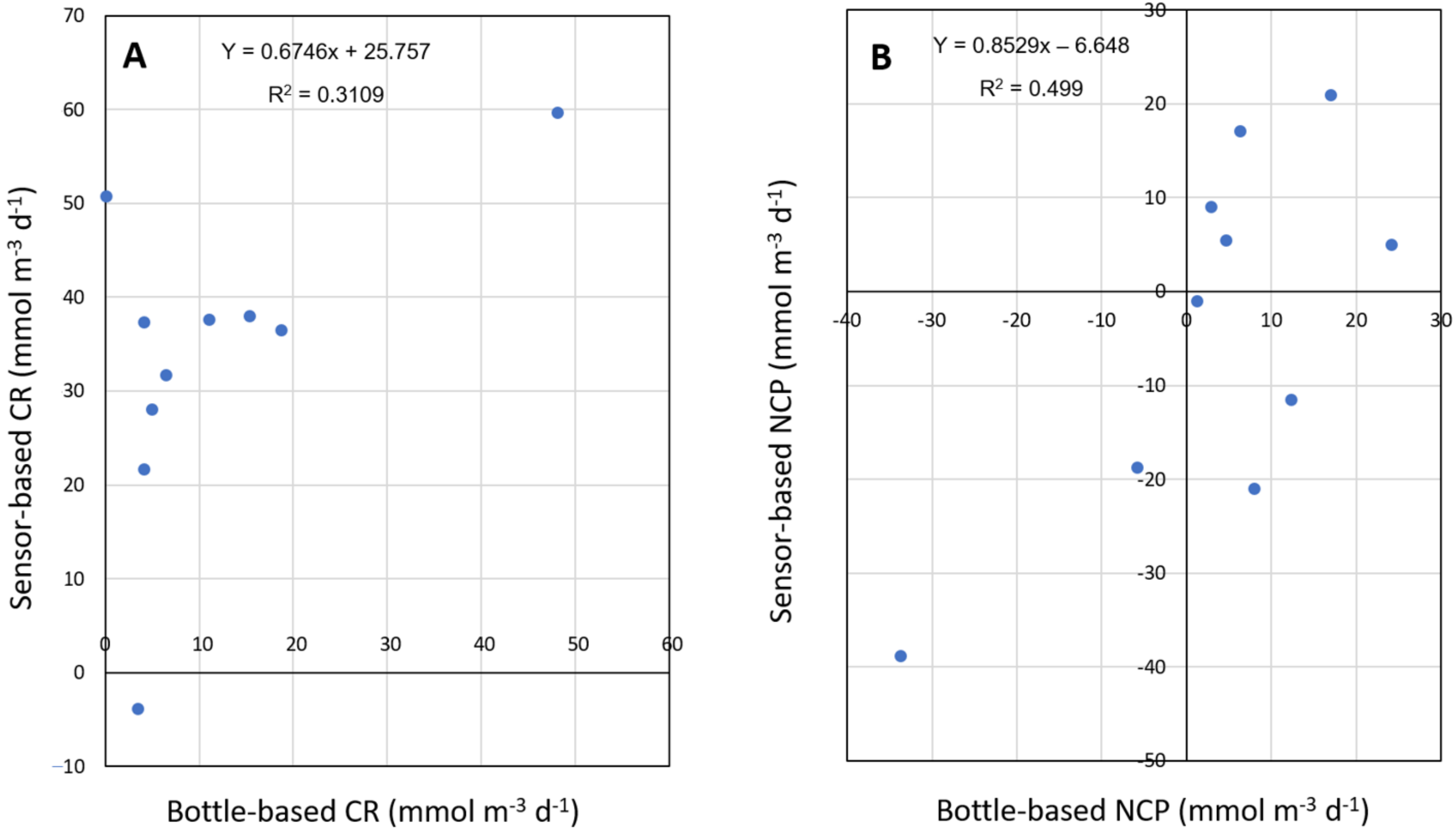


**Figure S3.** Comparison of *in situ* sensor-based rate calculations using the Slope Approach (DO concentrations versus time, logged every 10 minutes from 19:00 to 5:00, was used to calculate daily CR for each depth; daily NCP for each depth was calculated by adding half of the slope of daily CR to half of the slope from DO concentrations logged every 10 minutes from 7:00 to 17:00 and converted to a daily timescale). Bottle incubations took place in both meromictic (stratified) lakes: Ngermeuangel Lake (abbreviated NLK), Ongeim'l Tketau (OTM aka Jellyfish Lake), and T Lake (TLN); and holomictic (mixed) lakes: Heliofungia Lake (HLO), Mekeald Lake (MLN), Ngeruktabel Lake (NLN), and Uet era Ngchas (ULN). P=0.09, $r^2$=0.311 for CR and P=0.024, $r^2$=0.499 for NCP [n=10]. The outliers for CR ([-0.30, -50.68] and [-3.57, 3.88]) included two holomictic lakes (HLO and ULN) with strong connections with the outside ocean and extremely variable tidal fluxes.

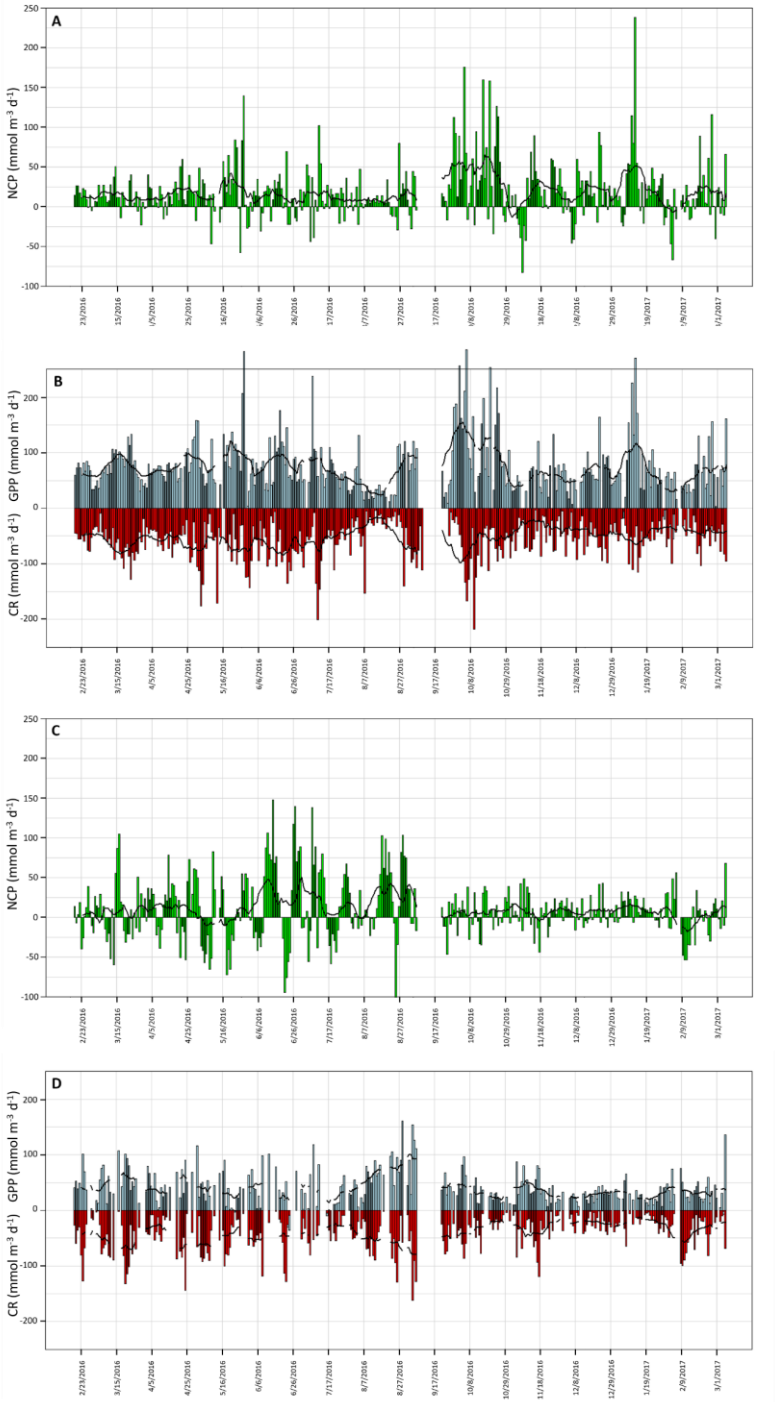


**Figure S4.** Daily rates at 1m and 6m with the 14-day moving average plotted on top for (A) NCP at 1m, (B) GPP and CR at 1m, (C) NCP at 6m, and (D) GPP and CR at 6m.

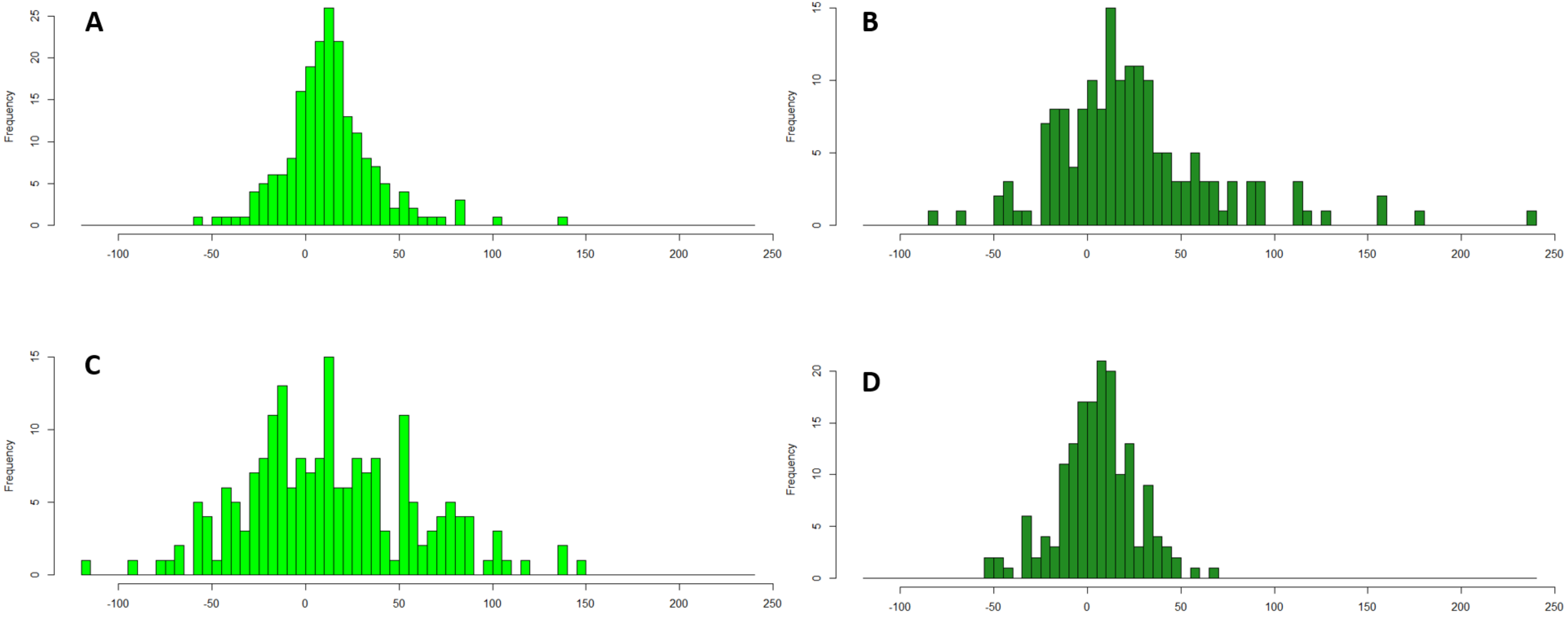


**Figure S5.** Histograms showing daily NCP at (A) 1m from 2/18/2016 to 9/6/2016, (B) 1m from 9/21/2016 to 3/6/2017, (C) 6m from 2/18/2016 to 9/6/2016, and (D) 6m from 9/21/2016 to 3/6/2017.
